# Significance of ARID1A in neuroblastoma onset mechanism

**DOI:** 10.3389/fonc.2025.1624561

**Published:** 2025-09-10

**Authors:** Duo Jin, Dong Zhang, Qinyu Tang

**Affiliations:** ^1^ Comprehensive Laboratory, The First People’s Hospital of Changzhou, Changzhou, China; ^2^ Comprehensive Laboratory, The Third Affiliated Hospital of Soochow University, Changzhou, China; ^3^ The Ruminant Disease Early Warning, Prevention and Control Technology Innovation Team, Institute of Special Wild Economic Animals and Plants, Chinese Academy of Agricultural Sciences, Changchun, China; ^4^ Department of Dermatology, The Second People’s Hospital of Changzhou, the Third Affiliated Hospital of Nanjing Medical University, Changzhou, China

**Keywords:** ARID1A, neuroblastoma, BAF complex, MYCN, treatment

## Abstract

Neuroblastoma (NB) is a prevalent pediatric malignancy with poor clinical outcomes. As one of the most common childhood malignancies, it can arise in various locations along the sympathetic nervous system, complicating both fundamental studies and therapeutic approaches. The ARID1A protein has emerged as a pivotal regulator in the pathogenesis of diverse tumor types within oncology research. Recent studies have increasingly focused on the functional role of ARID1A in NB pathogenesis. As a tumor suppressor, ARID1A loss-of-function mutations enhance migratory and invasive capacities of NB cells through cell cycle dysregulation, thereby promoting tumor cell proliferation. At the molecular level, ARID1A functions as the core subunit of the BAF chromatin remodeling complex, critically regulating the proliferative behavior of tumor cells. Although research in this field remains at an early stage, it has established a solid foundation for elucidating NB pathogenesis, with promising implications for improving clinical outcomes and quality of life in affected children. This review summarizes the critical role of ARID1A in NB and explores emerging therapeutic strategies, with particular emphasis on targeted protein degradation approaches and immunotherapeutic interventions.

## Introduction

Neuroblastoma (NB) is the fourth most common pediatric tumor, and affects 25–50 persons per million and accounts for 10-20% of pediatric malignancy related mortality (1). It can grow anywhere in the sympathetic nervous system since it affects neural crest-derived cells (2). NB may originate from multiple neural crest-derived cell types (e.g., neuroblasts, chromaffin cells, or mesenchymal-like cells) when genomic and epigenetic defects disrupt their normal differentiation during development (3). The precise moment and the specific cell or cells from which it arises remain uncertain and are still being investigated (4). The heterogeneity of NB is also reflected in the 5-year survival rate. Favorable NB, particularly low- and intermediate-risk cases, may spontaneously regress or differentiate without intervention, demonstrating 90-95% survival rates. Even with two novel therapies (anti-GD2 immunotherapy and tandem autologous stem cell transplantation), 40-50% of patients remain unresponsive to conventional treatments including surgery, chemotherapy, and immunotherapy (5, 6).

The BAF complex uses ATP hydrolysis to remodel nucleosomes and regulate gene expression (7). ARID1A serves as a core structural subunit of the BAF complex, mediating critical interactions with SMARCA4 and the base module to maintain complex integrity (8). Shuang He (8) et al. demonstrated that ARID1A deficiency disrupts BAF complex folding, significantly impairing nucleosome remodeling activity.

ARID1A recurrent mutations exhibit a broad tumor distribution, with particularly high prevalence in endometrioid and clear-cell ovarian cancers, gastric cancer, bladder cancer (9, 10) and neuroblastomas(~6%) (11, 12). Most ARID1A mutations are inactivating (frameshift or truncation) and distributed across the entire gene. These loss-of-function mutations are common in cancer, mainly including frameshifts, nonsense mutations, rearrangements, truncations, and splice-site defects (13). Emerging evidence indicates that ARID1A mutations serve as an independent prognostic biomarker, with loss-of-function variants significantly associated with reduced cancer-specific survival, increased recurrence rates (9) and diminished therapeutic response (14).

ARID1A has emerged as a critical tumor suppressor in cancer research. Although its role in NB remains poorly characterized, elucidating ARID1A-NB interactions may provide important insights into NB pathogenesis. This review summarizes current evidence on ARID1A in NB and discusses potential ARID1A-targeted therapeutic strategies.

## Mutations in ARID1A cause increased migration and invasion in NB

The association between ARID1A and NB was first reported in 2001 by Takeuchi et al. (15), who identified amplified expression of a truncated ARID1A isoform (SMARCF1/B120) in NB. Their immunohistochemical analysis of 23 NB cases demonstrated detectable expression of this truncated protein. The B120 gene product (truncated ARID1A) showed significant cytoplasmic and nuclear expression in 4/23 NB cases. However, these findings received limited attention, and *ARID1A/ARID1B* were still considered novel neuroblastoma-associated genes as late as 2013. Genomic analyses (whole-genome sequencing, rearrangement mapping, and exome sequencing) identified *ARID1A/ARID1B* deletions or mutations in 8/71 pediatric NB cases, which correlated with early treatment failure and poorer survival (11). This study further analyzed the functional roles of ARID1A and ARID1B. Notably, *ARID1B* primarily exhibited deletion mutations, whereas *ARID1A* showed point mutations or compound mutations with biallelic deletions. In 2020, Flora Cimmino et al. (16) developed a novel NGS (next generation sequencing) approach to identify the special change in cDNA of NB patients, which included changes in the *ARID1A* gene. This role of *ARID1A* was further affirmed in 2023 by Zekiye Altun (17) et al. Through whole-exome sequencing and analysis using the cBioPortal cancer genomic database, their study identified *ARID1A* as one of two key high-risk genes.

C. Li (18) et al. demonstrated that *ARID1A* knockdown in SK-N-SH NB cells using shRNA significantly enhanced cell proliferation. Flow cytometry analysis confirmed cell cycle dysregulation, showing decreased G0/G1 phase and increased S phase populations. These results demonstrate that *ARID1A* knockdown disrupts cell cycle progression. Meanwhile, wound healing and transwell assays showed *ARID1A* silencing significantly increased cell migration rates and enhanced invasive capacity. Extensive studies have established ARID1A as a high-risk factor in NB pathogenesis. Multiple experimental approaches have consistently validated this finding. However, the molecular mechanisms underlying the regulation of ARID1A in NB remain unclear.

**Figure 1:**
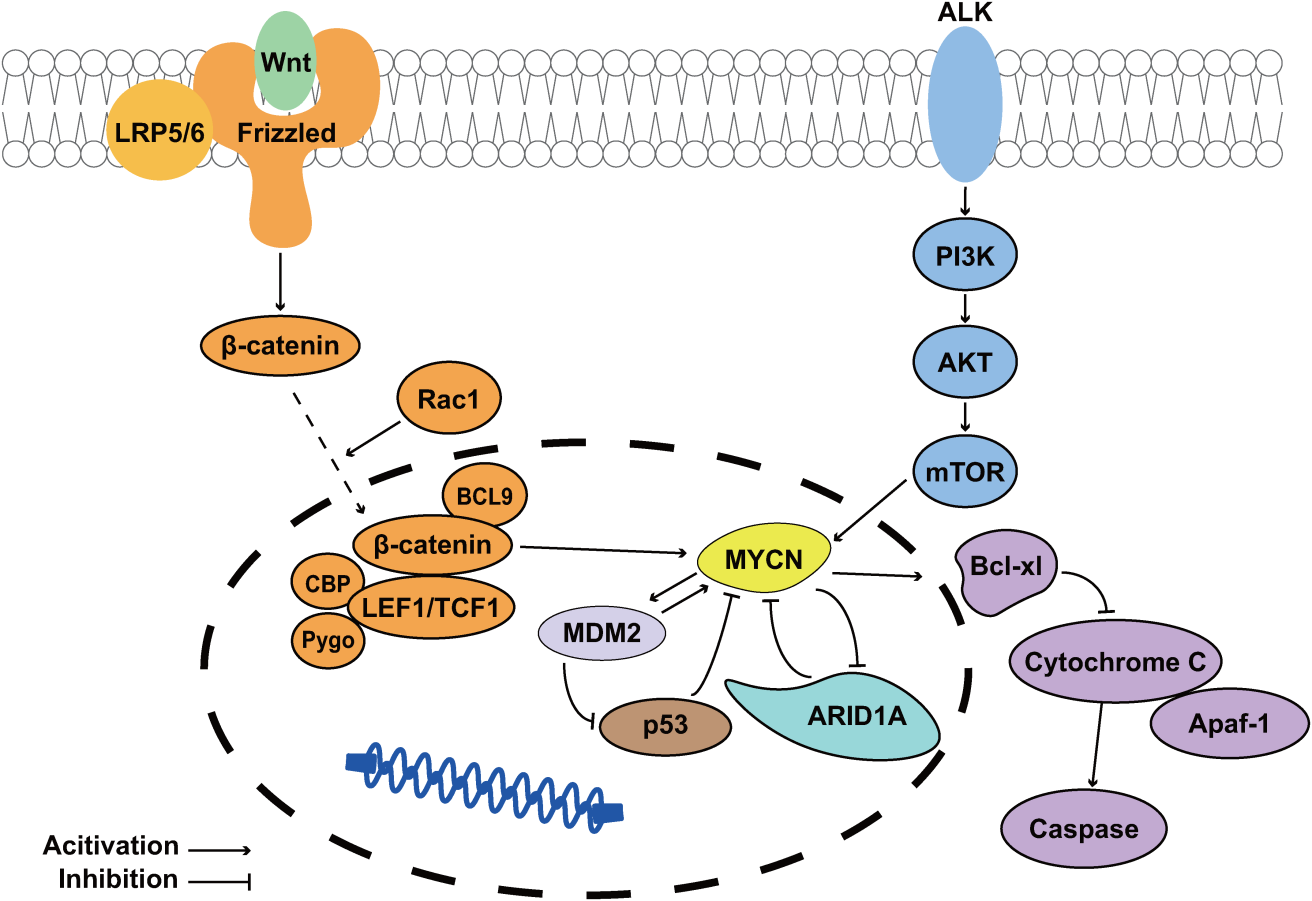
*ARID1A* acts as a tumor suppression gene of *MYCN*-driven NB. The PI3K/AKT/mTOR and Wnt signaling pathways upregulate *MYCN* expression, playing crucial roles in NB cell survival and drug resistance development. *MDM2* suppresses the tumor suppressor *p53* while simultaneously enhancing *MYCN* expression (24); The *MYCN*-encoded protein N-Myc transcriptionally upregulates Bcl-xL, thereby inhibiting apoptosis in NB cells (25, 26); Notably, *ARID1A* functions as a *MYCN* suppressor, and its loss promotes *MYCN*-driven NB pathogenesis.

Chromosomal instability (CIN) is implicated in 90% of malignant tumors, with increasing severity correlating with worse prognosis (19). The 1p36 locus is among the most frequently deleted chromosomal regions across diverse malignancies, including neural, epithelial, and hematopoietic cancers. The 1p36 deletion exhibits a strong predilection for neurological tumors, with particularly high prevalence in NB (20). Notably, comprehensive genomic analyses reveal that approximately 70% of *MYCN*-driven NB cases harbor 1p36 deletions (21). *MYCN* gene amplification and overexpression represents one of the most prevalent high-risk genetic alterations in NB, occurring in approximately 25% of cases (22). Jesus García-López et al. (21) demonstrated through *in vitro* transformation assays using primary mouse neural crest cells (NCCs) that *MYCN*-driven carcinogenesis differentially transforms NCCs with ARID1A deletion compared to those with random 1p36 deletions, thereby identifying *ARID1A* as a *MYCN*-specific tumor suppressor. Hui Shi et al. (12) employed a zebrafish model [Tg(dβh:EGFP-MYCN)] to investigate the ARID1A-MYCN interaction in NB, wherein the dβh promoter drives EGFP-MYCN fusion gene expression for tumor visualization. *ARID1A* knock-down mutations were detected in early-onset tumors (5–13 weeks post-fertilization, wpf) but not in late-onset tumors (15–27 wpf). These results demonstrate that *ARID1A* loss-of-function advances the onset of MYCN-driven NB by 10–14 weeks in the *Tg(dβh:EGFP-MYCN)* model. Therefore, the deletion of ARID1A and overexpression of MYCN are synergistic in NB development and also imply a tumor suppressive role of ARID1A in NB (**Figure 1** ARID1A acts as a tumor suppression gene of *MYCN-driven* NB.). Furthermore, RNA sequencing (RNA-seq) and Western blot analyses, combined with Transwell invasion assays, revealed that *ARID1A* depletion in MYCN-driven NB induces a phenotypic shift from the differentiated adrenergic (ADRN) state to a more aggressive mesenchymal (MES) state, as evidenced by altered expression of lineage-specific markers and enhanced tumor invasiveness (12, 23). In summary, these findings establish a critical functional interplay between *ARID1A* and *MYCN* in NB pathogenesis. Specifically, *ARID1A* acts as a tumor suppressor, and its loss exacerbates malignant progression by enhancing tumor cell invasiveness and metastatic potential. However, as a core subunit of the BAF chromatin remodeling complex, the mechanistic contribution of *ARID1A* within this complex to NB biology remains poorly understood.

## Disruption of the BAF complex in which ARID1A resides inhibits NB proliferation

The BAF chromatin remodeling complex plays critical roles in tumorigenesis across multiple cancer types. As ARID1A is an essential subunit of this complex, an important question arises: To what extent can the functional consequences of ARID1A alterations be extrapolated to the ARID1A-containing BAF chromatin remodeling complex. Bieke Decaesteker et al. (27) found that SOX11 regulated 10 core proteins of the BAF complex, including ARID1A. Their findings indicate that the observed suppression of NB invasiveness and metastatic potential resulted from structural destabilization of the BAF complex, mediated through SOX11-dependent silencing of both its essential subunits and direct target ARID1A. This result differs from the effects observed with ARID1A as an individual component. Since SOX11 regulates 10 subunits of the BAF complex besides ARID1A, the disparity in conclusions might stem from strong alterations in the remaining subunits. It is also possible that the BAF complex, under the synergistic effect of multiple genes, exerts effects inconsistent with those of ARID1A alone. Alterations in the remaining 9 subunits could potentially lead to disruption of the integrity of the BAF complex and subsequently the balance of regulation between individual subunits.

**Figure 2:**
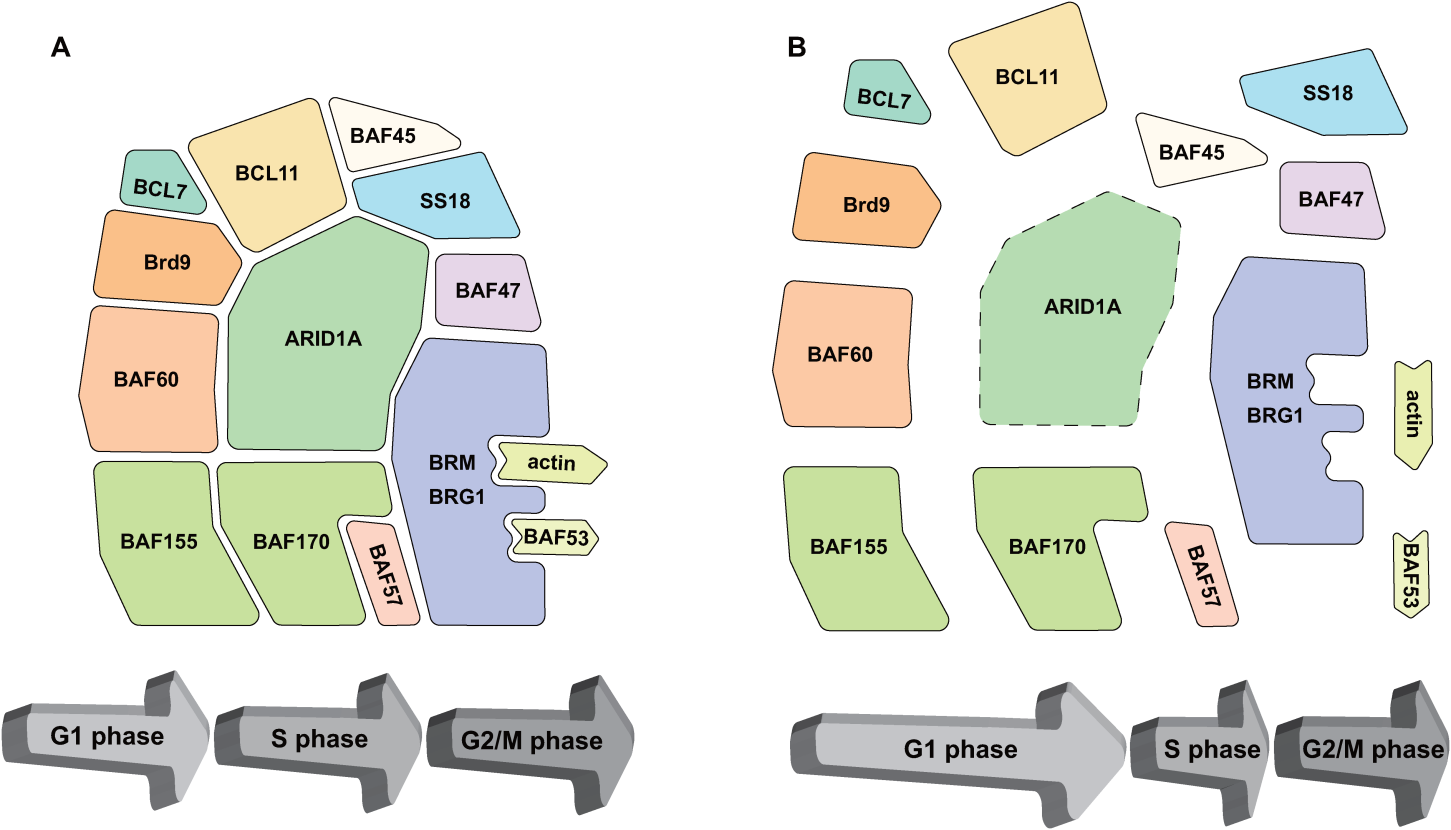
Targeting of ARID1A results in disassembly of the BAF complex, affecting the cell cycle of NB cells. **(A)** Complete BAF Complex and normal cell cycle. **(B)** The disintegration of the BAF complex is accompanied by an abnormal cell cycle mainly characterized by the prolongation of the G1 phase.

It is hypothesized that this outcome is attributed to the disruption of the integrity of the BAF complex. Another study conducted in 2022 lends support to this hypothesis (28). Silencing the BAF-specific subunits ARID1A and ARID1B simultaneously reveals that NB cell proliferation depends significantly on the structural integrity of the BAF complex. In NB cells, this perturbation markedly reduced cyclin D1 protein levels and Rb phosphorylation, while flow cytometry analysis confirmed significant G1-phase accumulation with concomitant decreases in S and G2/M-phase populations. BAF complex disruption induced transcriptome-wide reprogramming in NB cells, altering cell cycle regulators and triggering G1-phase arrest, ultimately suppressing proliferation (**Figure 2**) (28). A 2023 study by Katerina Cermakova (29) et al. provided further evidence that the BAF complex is essential for maintaining adrenergic core regulatory circuitries (CRCs) in NB, primarily by preserving the open chromatin state of G1-phase enhancers and activating cell cycle-related genes. Inactivation of the BAF complex severely impairs the G1-to-S phase transition, leading to G1 arrest and ultimately cell death. These findings demonstrate that the BAF complex, as an integrated unit, differs markedly from ARID1A in both its mechanistic actions and phenotypic outcomes, underscoring the unique role of ARID1A in NB pathogenesis and highlighting its significance as a critical protein warranting further investigation.

While the architecture of the BAF complex has been partially characterized, structural insights into the ARID1A-deficient model remain elusive, while the structure of the BAF complex has been partially characterized, structural insights into the ARID1A-deficient model remain elusive. Furthermore, neither the standalone structure of ARID1A protein nor its interactive architectures with other proteins in NB have been resolved.Current structural data of the intact BAF complex lack complete information about ARID1A as well (8), fundamentally limiting our understanding of its mechanistic role. Future studies should employ cryo-EM and other structural biology approaches to determine how ARID1A deficiency alters the complex’s conformation and functional properties.

## Future research directions at the therapeutic strategies in ARID1A-mutant NB

Most studies associate ARID1A loss with poor prognosis and tumor progression, recent studies suggest it may also increase susceptibility to specific therapies. Gene promoter hypermethylation correlated with reduced *ARID1A* expression in 86.4% of invasive ductal breast carcinomas (30). Decreased ARID1A immunoreactivity was associated with poorer prognosis in breast cancer patients. Both complete loss and partial reduction of *ARID1A* expression correlated with more aggressive tumor behavior. Furthermore, ARID1A protein expression served as an independent prognostic marker, with higher levels predicting better clinical outcomes (31).

Although ARID1A mutation is not an initiating event in NB, its deletion promotes disease progression and correlates with poor prognosis. Notably, immunoprecipitation assays using anti-ARID1B antibodies demonstrated that ARID1B-containing BAF complexes remain structurally intact in ARID1A-deficient NB cells (12). Consistent with prior studies demonstrating synthetic lethality between ARID1B and ARID1A, genetic depletion of ARID1B in ARID1A-deficient cells destabilizes the BAF complex and impairs cellular proliferation. These findings suggest ARID1B as a potential therapeutic target, though ARID1A loss itself remains clinically detrimental in NB patients. Recent advances in Proteolysis Targeting Chimeras (PROTACs) have demonstrated successful protein degradation strategies (32), including targeted disruption of the BAF complex. Notably, PROTAC-mediated degradation of the ATPase subunits has shown therapeutic efficacy in non-small cell lung cancer (33) (NSCLC) and prostate cancer (34). In NB therapeutic research, Adam D Durbin (35) et al. employed PROTAC technology to selectively degrade EP300 as a treatment strategy for MYCN-amplified NB. These findings provide a strong rationale for exploring ARID1B degradation in ARID1A-deficient NB, which may suppress tumor proliferation by destabilizing the BAF complex.

Beyond targeted protein degradation, emerging therapeutic strategies are now focusing on aberrantly expressed circular RNAs (circRNAs) in tumor cells. Aberrant expression of circRNAs has been implicated in nearly all cancer types, where they function as either oncogenes or tumor suppressors during tumorigenesis (36). In NB, circRNAs exhibit dual roles in tumor suppression and oncogenesis (37). Specifically, circARID1A has been shown to promote tumor cell proliferation and survival (23). Current therapeutic strategies targeting circRNAs include targeted degradation (38), immunotherapy (39), and RNA vaccine-based approaches to enhance treatment efficacy (40). However, research on circRNA-targeted therapies remains in its early stages, warranting further investigation.

Dysregulation at both the protein and RNA levels in tumor cells is fundamentally sustained by the tumor microenvironment (TME). TME not only provides structural support for tumor growth but also serves as a critical platform for complex cellular signaling interactions that collectively drive tumor proliferation and invasion. Within this context, immunotherapy has emerged as a promising strategy to enhance cancer treatment efficacy by modulating the immunosuppressive TME (41). Guangsheng Zhu (42) et al. conducted a large-scale clinical analysis of NSCLC patients and demonstrated that those harboring ARID1A/ARID1B mutations exhibited enhanced clinical responses to immune checkpoint blockade therapy, including PD-1/PD-L1 and CTLA-4 inhibitors. In NB treatment, immune checkpoint blockade therapy has been reported (43). Studies indicate that PD-L1 expression correlates with *MYCN* amplification, while ARID1A-mutated NB is closely associated with *MYCN* status. Immune checkpoint therapy may be particularly effective in ARID1A-mutated NB, provided these tumors exhibit PD-L1 expression. In a cohort of 31 NB patients analyzed by Shogo Zuo (44) et al., PD-L1 expression was detected in 11 cases (35%). These PD-L1-positive tumors may represent a promising target for immunotherapy. However, this study did not assess how many of the 11 PD-L1-positive cases harbored ARID1A mutations, nor whether such mutations would yield therapeutic outcomes similar to those observed in NSCLC. Thus, this potential treatment strategy remains theoretical at present.

The therapeutic potential of these approaches in NB may be further limited by poor drug delivery across the blood-brain barrier (45). To address these challenges, nanoparticle-based drug delivery systems (46, 47) and nanomedicines (48) have emerged as promising approaches. These technologies enhance tumor-specific drug accumulation, prolong drug retention, and minimize systemic toxicity (47). Integrating nanotechnology with existing therapies may thus provide a synergistic strategy to improve NB treatment outcomes.

## Conclusions

ARID1A, a core subunit of the BAF chromatin remodeling complex, plays a pivotal role in NB pathogenesis. Studies have demonstrated that ARID1A synergizes with *MYCN* amplification to drive the transition of tumor cells from an adrenergic to a mesenchymal phenotype, thereby enhancing invasive and migratory capacities and accelerating disease progression. However, the role of ARID1A within the regulatory network of NB pathogenesis remains incompletely understood and warrants further investigation. Based on existing research findings, it is hypothesized that ARID1A may suppress *MYCN* transcription or inhibit its transcriptional activation function at the genetic level, thereby disrupting *MYCN*-driven signaling networks during NB initiation and progression. This proposed mechanism would position ARID1A as a tumor suppressor in this context. However, the specific involvement of ARID1A in *MYCN*-driven NB signaling networks has not yet been experimentally validated. Although the structures of the BAF complex and several related family members were resolved around 2020, the structural consequences of ARID1A deletion remain unknown. The lack of information on the conformational state of the ARID1A-deleted BAF complex and its associated structural rearrangements presents a critical knowledge gap, hindering mechanistic understanding at the molecular level.

Despite recent advances, several critical gaps remain in understanding the mechanistic role of ARID1A in NB. First, the cooperative interaction network between ARID1A and MYCN requires further elucidation, particularly regarding the functional involvement of the BAF complex in mediating their crosstalk. Second, therapeutic strategies targeting ARID1A-deficient NB remain underdeveloped. While several potential approaches have been proposed, most are still in the conceptual or preclinical stage, and their clinical efficacy awaits rigorous validation. Integrating clinical investigations with mechanistic studies will be essential to establish a solid theoretical foundation and identify actionable therapeutic pathways for ARID1A-driven NB.
